# The influence of AI on the economic growth of different regions in China

**DOI:** 10.1038/s41598-024-59968-7

**Published:** 2024-04-22

**Authors:** Shuang Lin, Minke Wang, Chongyi Jing, Shengda Zhang, Jiuhao Chen, Rui Liu

**Affiliations:** 1 School of Economics and Management, Civil Aviation Flight University of China, Deyang, 618307 China; 2 School of Airport Engineering, Civil Aviation Flight University of China, Deyang, 618307 China; 3 Department of Administration, Chengdu University of TCM, Chengdu, 611137 China

**Keywords:** AI technologies, China, Regional economic development, Environmental social sciences, Environmental economics

## Abstract

High-quality development plays a crucial role in China’s economic progress in the new era. It represents a new concept of advancement and mirrors the increasing aspirations of the populace for an improved standard of living. In this context, the role of artificial intelligence (AI) in promoting sustainable development cannot be overemphasized. This paper explores how AI technologies can drive the transition to a green, low-carbon and circular economy. We have established an index system to measure the development level of the artificial intelligence industry and the high-quality development of the economy, which is relevant to the current state of the artificial intelligence industry and the advancement of the economy. Panel data from 2008 to 2017 has been utilized for this purpose. Global principal component analysis method and entropy value method are employed in the evaluation. Through in-depth analysis of the application of artificial intelligence and environmental protection in various provinces and cities, we clarify that artificial intelligence promotes innovation, saves resources, and is conducive to the development of green economy in the new era.

## Introduction

The world is facing unprecedented environmental challenges, from rising carbon emissions to the depletion of natural resources, which remind us that we must take action to change^1,2^. Adopting sustainable practices has become a priority for governments, businesses and individuals. At the intersection of technology and sustainability, AI has become a transformative force, offering unprecedented opportunities to advance a green, low-carbon and circular economy. In recent years, the rapid advancement and extensive utilization of artificial intelligence technology have led to the establishment of an industrial chain known as “artificial intelligence +”, giving rise to fresh formats, models, and directions for industrial development. Artificial intelligence serves as a crucial driving force behind a new wave of scientific and technological revolution and industrial transformation^3–5^. It can be seen that in the new round of information technology revolution, the artificial intelligence industry will become a new driving force for promoting high-quality economic development. At present, the new generation of artificial intelligence industry includes three levels: base layer, technology layer and application layer, and has a complete industrial chain. Its advantages are mainly reflected in: First, a number of technologies in the field of artificial intelligence industry technology layer are at the international frontier level; Second, the artificial intelligence industry occupies a huge market in the application layer field and has the support of information data. Promoting the development of artificial intelligence industry is an important way to accelerate innovation-driven development and industrial transformation and upgrading, and is a new driving force for reinforcing the advancement of superior economic growth^6,7^. At the same time, it is also the best choice to reduce environmental pollution and develop green and low-carbon economy^8^. At present, under the background of global economic development and the continuous improvement of international technological level, artificial intelligence technology and intelligent industry are entering a period of rapid development. Studies have pointed out that the scale of China's AI industry has reached 10.06 billion in 2018, with a growth rate of 43.3%, and will grow by 3.43 billion in 2022^9,10^. This indicates that there is significant potential for the development of China’s artificial intelligence industry. Currently, artificial intelligence technology and industry are rapidly integrating and developing, leading to the expansion of industrial chain layout, the formation of industrial chain clusters, and the promotion of transformation and upgrading in traditional industries as well as high-quality economic development. Based on this, China has incorporated artificial intelligence into the national strategic level, and attaches great importance to intelligent technological progress and industrial development^11,12^.

This paper uses a dynamic panel model to conduct an empirical study on how the artificial intelligence industry impacts the high-quality development of the green economy. The analysis takes into account the dynamic nature of economic growth and addresses endogenous problems caused by missing variables. This research provides a theoretical foundation for our country to promote high-quality economic development through the “artificial intelligence + X” initiative, thus holding certain theoretical value.

## Literature review

Foreign scholars started the research on artificial intelligence and industrial development earlier, and the existing research literature mainly focuses on the connotation of artificial intelligence, the existing problems and risks of artificial intelligence development, application prospects, and technological innovation. First of all, there are different interpretations about the meaning of artificial intelligence, and no unified definition has been given. Minsky pointed out that artificial intelligence is a complex science that replaces human intelligence; Min also believes that AI is a “thinking machine” developed and created, which can accurately imitate, learn, and replace human intelligence^13,14^. Secondly, as the theoretical connotation of artificial intelligence continues to improve and expand, its technology has a significant impact on the development of various industries and overall economic operation. The influence of artificial intelligence on industrial development is examined through the lens of its technological applications. The research of Mian^15^ shows that artificial intelligence can not only promote the exchange of information in the industrial supply chain, but also replace assets with information elements, reduce unnecessary transaction costs, and help enterprises improve production and operation efficiency. Based on the development theory of artificial intelligence, Frey and Osborne^15^ took the study of the degree of response of intelligent machines to work as an entry point and found that the American labor market would change unpredictably due to intelligent technology.

In recent years, there has been a gradual increase in the development of domestic artificial intelligence and industry. Scholars have primarily focused on the national policy context for the development path of artificial intelligence, its application fields, and quantitative analysis. Firstly, considering the support of national policies, discussions have been made on the choice of social governance path in the era of artificial intelligence, as well as the impact of human intelligence technology on industrial transformation and employment. Mei^16^ pointed out that in this era of intelligence, it is important to effectively utilize intelligent technology to enhance national governance efficiency and mitigate any negative effects on employment caused by artificial intelligence. Xie^17^ pointed out that the scale of China’s AI industry is gradually expanding, the artificial intelligence service platform is becoming more and more perfect, and the industrial development layout of Shanghai, Guangzhou and Shenzhen is formed in the north.

## Materials and methods

The research methods of this paper mainly include^19,20^:

1. The use of logical reasoning methods. In exploring the mechanism analysis of artificial intelligence industry on high-quality economic development, the method of logical reasoning is used to analyze the internal impact of human intelligence industry and high-quality economic development from the level of economic effect, environmental effect and social effect.
2. Using statistical techniques, this paper employs the global principal component analysis method and entropy value method to assess the development level of the artificial intelligence industry and the high-quality development level of the economy. The evaluation and instruction system is constructed to measure the artificial intelligence industry development index and economic high-quality development index of various provinces in China from 2008 to 2022. The region and time characteristics of the index variables are depicted using statistical software to create tables in multiple dimensions.
3. Using econometric methods. In the empirical part of this paper, the dynamic panel model is mainly constructed to explore the research and analysis of the influence of artificial intelligence industry on high-quality economic development. At the same time, under the premise of considering the endogenous problem, the panel fixed effect and panel random effect measurement methods are adopted, and the system generalized moment estimation method (GMM) is used for regression^18–21^. At the same time, when analyzing the data stationarity, the unit root stationarity test is used.
4. Artificial intelligence industry index selection and data sources. The data of the artificial intelligence industry development index and high-quality economic development index involved in the econometric model come from the statistical data and related research of the national statistical database, EPS database, and China Economic network database.
5. Artificial intelligence industry index measurement. Although the selection of artificial intelligence industry index construction considers the development of artificial intelligence industry from different aspects, there is still a correlation between various indicators. Therefore, in order to further reduce the correlation among indicators, this paper adopts the global principal component analysis method to combine basic support, integrated application, innovation ability and environmental protection into a comprehensive index, namely artificial intelligence industry index.

## Results

Artificial intelligence industry is a very broad field, it refers to the artificial intelligence technology as the core, by the foundation support and application integration of the industry. Basic support is mainly composed of information data and calculation ability support, which provides basic elements for the development of artificial intelligence technology and its industry; Integration specifically refers to various subsectors including intelligent finance, and robotics. From the industrial level, the artificial intelligence industry: one is the industry with artificial intelligence technology as the core. Such industries directly provide products and services through artificial intelligence technology itself, effectively improving the efficiency of industrial operations. The other is the artificial intelligence technology integration industry. This kind of industry is mainly through the innovative integration of artificial intelligence technology and traditional industries, so as to upgrade traditional industries and form new intelligent industries. These two types of artificial intelligence industries are based on artificial intelligence technology as the core element of development, with intelligent agglomeration, data-driven, information sharing and other characteristics.

### Artificial intelligence industry index selection and data sources

The data used in this paper is primarily sourced from various Chinese statistical yearbooks covering the period from 2008 to 2022, including the China Statistical Yearbook, China High-tech Industry Statistical Yearbook, China Science and Technology Statistical Yearbook, and China Electronic Information Industry Statistical Yearbook.

### Artificial intelligence industry index measurement

Although the selection of artificial intelligence industry index construction considers the development of artificial intelligence industry from different aspects, there is still a correlation between various indicators. Therefore, in order to further reduce the correlation between indicators, this paper adopts the global principal component analysis method to combine these 15 indicators into a comprehensive index, namely, artificial intelligence industry index. In this paper, the SPSS22 econometric and statistical software is used to conduct principal component analysis on the AI industry development indicators of 30 provincial cities in China from 2008 to 2022, and then the AI industry development index is constructed.

The test results of this paper show (Table 1) that the appropriate measure value of KMO sampling is 0.72, greater than 0.6, which meets the prerequisite of principal component analysis, indicating that the artificial intelligence industry index data is suitable for principal component analysis (*p* < 0.05). Determine the principal component factors. According to the theoretical experience of principal component analysis, it is more appropriate for the component with eigenvalue greater than 1 to be the principal component. When the eigenvalue is greater than 1, only the first two components are available, and the cumulative variance contribution rate is 82.03%, indicating that these two components can fully reflect the information of the original data. Therefore, we extract these two components as the main components and record them as F1 and F2 respectively.

**Table 1:** KMO test and Bartlett sphericity test results.

KMO test		0.72
Bartlett sphericity test	Chi square	9567.87
	df	120
	*p*	0.000

Regression estimation method was used to calculate the factor score coefficient and the weight of each index, see Table 2.

**Table 2:** Factor score coefficient and weight result.

Name	F1	F2	Coefficient	Weight (%)
S1	0.2924	− 0.1212	0.1301	6.76
S2	0.2398	− 0.0324	0.2344	6.32
S3	0.2774	− 0.3943	0.0985	7.19
S4	0.2003	0.4839	0.0075	4.29
S5	0.2437	− 0.3839	0.2056	5.87
S6	0.3209	− 0.3232	0.2058	6.34
S7	0.3112	− 0.1249	0.1960	7.29

Determine the artificial intelligence industry index. According to the results of principal component analysis, the development index of artificial intelligence industry is determined. Artificial intelligence industry (AI) is used to represent the artificial intelligence industry, and the artificial intelligence industry index is expressed as^14^:

1 $$ {\text{AI}} = 0.1301{\text{S}}1 + 0.2344{\text{S}}2 + 0.0985{\text{S}}3...... + 0.1960{\text{S}}7 $$

According to previous research, the development level of artificial intelligence industry in each province is standardized:

2 $$AI=\frac{Fi}{maxFi-minFi}$$

where Fi is the comprehensive factor score of i province. The maxFi and min Fi are the maximum and minimum values of comprehensive factor scores corresponding to province i.

### Calculation results and analysis of artificial intelligence industry indicators

**Figure 1:**
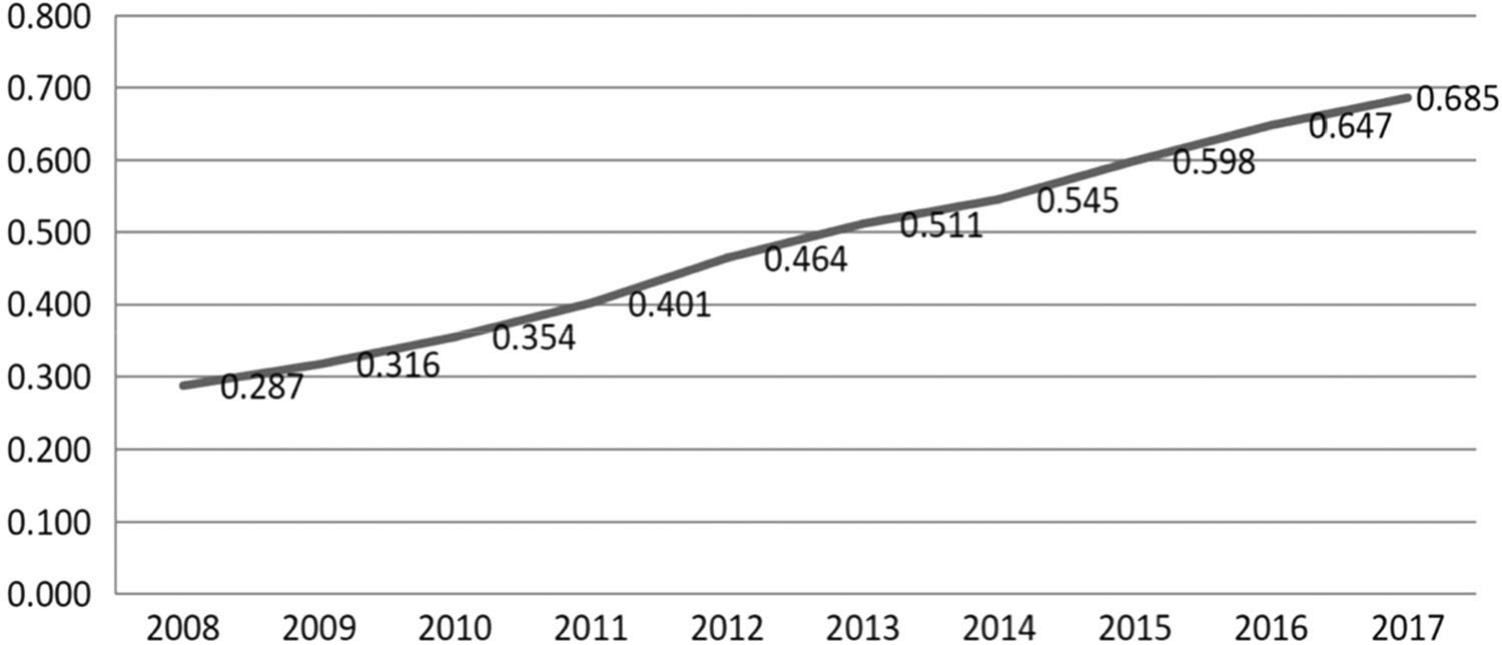
National average artificial intelligence industry development level from 2008 to 2017.

From the perspective of the overall change characteristics: from 2008 to 2017, with the comprehensive optimization of China’s artificial intelligence industry development policy, the steady improvement of innovation capacity, the continuous consolidation of the industrial foundation, the continuous improvement of the development environment, and the continuous deepening of integration and application, the development of artificial intelligence industry in 30 provinces in China showed a good trend of rising year by year. This is the result of the state’s strong support for the construction of intelligent industries, continuous promotion of artificial intelligence technology innovation and progress, and the rapid development of artificial intelligence industry in the whole society. Our research shows the artificial intelligence industry development index and its average national artificial intelligence industry during 2008–2017, and Fig. 1 shows the overall trend of the average national artificial intelligence industry development level during 2008–2017. China's artificial intelligence industry development index is 0.481, which is higher than the national average level of artificial intelligence industry development in 15 provinces, of which Beijing (1.033), Shanghai (0.946), Jiangsu (0.876), Guangdong (0.867) and Zhejiang (0.830) are ranked in the top 5 in China’s artificial intelligence industry development. Shanxi (0.189), Gansu (0.160), Xinjiang (0.159), Hainan (0.061), and Qinghai (0.052) are ranked in the bottom 5 of China, among which Hainan is divided into the eastern region, but its economic development and scientific research level are still a certain gap with other eastern regions, so the development level of artificial intelligence industry is low. This shows that the development trend of China's artificial intelligence industry is similar to that of economic development, and also shows a decreasing trend year by year from the southeast coast to the northwest inland.

**Figure 2:**
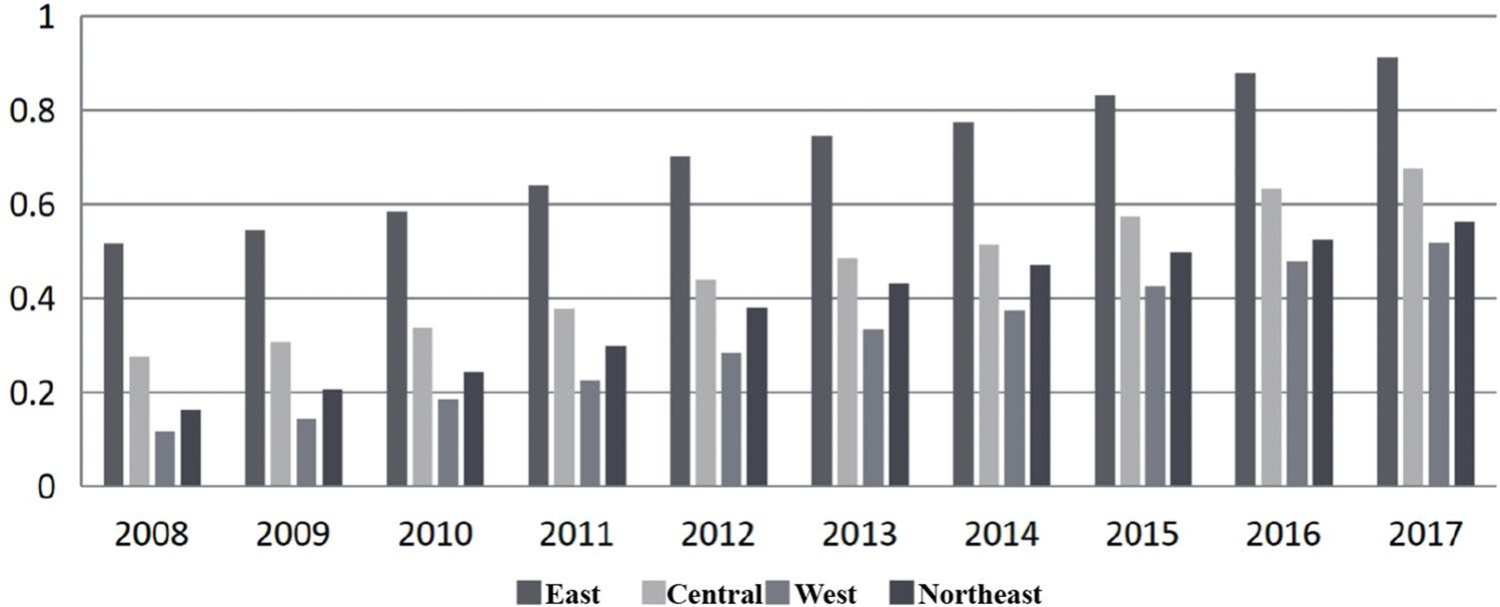
Change trend of sub-regional artificial intelligence industry development index (From left to right, East, central, West, Northeast of China).

From the perspective of regional fluctuation characteristics (as shown in Fig. 2): 1. The average development index of industrial intelligence industry is 0.713, exceeding the national average level of 0.232, and is in an absolute leading position. In the eastern region, Beijing and Shandong, relying on the construction of smart city clusters around the Bohai Sea economic Circle, have core competitive advantages in intelligent technology research and development and intelligent industry cultivation. Revised: Leveraging the manufacturing hub of the Yangtze River Delta Economic Belt, Shanghai, Jiangsu and Zhejiang have actively advanced intelligent manufacturing technologies such as the Internet of Things and cloud computing, driving widespread development of the artificial intelligence industry across various social and economic sectors. Building on the competitive edge of the Pearl River Delta Economic Circle and the Guangdong-Hong Kong-Macao Greater Bay Area, Guangdong will further enhance its strategic layout and construction in the artificial intelligence industry to achieve leading-edge development in this field. 2. Balanced development of the central region. The average development index of artificial intelligence industry in the central region is 0.462, and the development is balanced within the region. Among them, the development level of artificial intelligence industry in Anhui (0.587), Henan (0.523) and Hubei (0.563) is relatively close, and is in a higher position in the central region. 3 Polarization in the western region. The average index of artificial intelligence industry development in the western region is 0.309, lagging behind the national average level of 0.172, and the development in the region is significantly polarized. The combination of policies and industries in Sichuan and Chongqing has effectively promoted the rapid development of the artificial intelligence industry, and the artificial intelligence industry index ranks 9th and 16th in the country respectively. Shaanxi and Guizhou have outstanding advantages in science and technology research and development and big data application, accelerating artificial intelligence and industrial development. The development level of artificial intelligence industry in other western regions is relatively backward. 4. The level of northeast China is not high. The average development index of artificial intelligence industry in Northeast China is 0.378, higher than the level of western regions, but still lower than the national development level of 0.103. Among them, Liaoning’s artificial intelligence industry development index of 0.554 is higher than the national average, ranking first in Northeast China. The overall level of development of artificial intelligence industry in Northeast China is not high, which may be related to factors such as insufficient internal impetus for economic development in Northeast China, difficulty in converting old and new economic momentum, small scale of development of artificial intelligence industry, and insufficient investment.

This paper analyzes the impact of the artificial intelligence industry on high-quality economic development by incorporating the artificial intelligence industry development index into the framework of analysis. In order to account for the dynamic nature of high-quality economic development, we also include a first-order lag variable of the explained variable in our analysis.

3 $$ HQD_{it} = B_{0} + B_{1} HQD_{it - 1} + B_{2} AI_{it} + B_{3} X_{it} + E_{it} $$

In the above formula, t and i respectively represent different years and provinces; HQDit = the high-quality economic development index of the i province in the t year; *HQDit-1* = the high-quality economic development index of the i province in the one period lagging behind; *AIit* = the artificial intelligence industry development index of the i province in the t year; *Xit* = the control variable of the i province in the t year. B_0_ represents the intercept term, *Eit* = the random error term. *B*_1,2,3_ = parameter to be estimated, *B*_2_ = the influence coefficient of artificial intelligence industry on high-quality economic growth. Considering the economic meaning of regression coefficient, and in order to increase the smoothness of data, logarithmic processing is carried out on the variables of artificial intelligence industry index, economic high-quality development index and technological innovation.

The findings indicate that the artificial intelligence industry index, as the core explanatory variable, has a positive impact on high-quality economic development and passes the significance test at the 1% level. Economic development is a continuous process of gradual transformation.

## Discussion

At present, Chinese and foreign scholars have studied the relationship between artificial intelligence industry and economic growth, and scholars generally agree that artificial intelligence industry has a role in promoting economic growth. Although there is a wealth of existing literature on the influence of artificial intelligence on economic growth, there is a lack of systematic analysis in the literature regarding the impact of the artificial intelligence industry on the mechanism for high-quality economic development. This paper examines the economic, environmental, and social implications of the artificial intelligence industry on high-quality economic growth based on the concept of high quality^16,17^. After the introduction of China’s artificial intelligence industry development strategy, artificial intelligence has significantly contributed to enhancing the country's economic growth. Therefore, it is crucial to understand the impact of the artificial intelligence industry on high-quality economic development in order to further strengthen China’s overall artificial intelligence industry and promote high-quality economic growth driven by artificial intelligence. This study utilizes provincial panel data from 2008 to 2017 to construct a provincial index for artificial intelligence industry development and economic high-quality development, and empirically analyzes the influence of the artificial intelligence industry on high-quality economic development.

### The role of artificial intelligence industry in promoting innovation ability

The artificial intelligence industry improves its innovation ability by accelerating the introduction of intelligent technologies. In terms of optimizing traditional elements, the development of artificial intelligence industry can not only introduce high-end information technology elements such as cloud computing, new Internet, and big data, expand people's learning ability, and accelerate human capital accumulation, but also build industrial operation systems, improve technological innovation systems, and improve resource allocation efficiency under the information environment of digital economy operation. In terms of promoting technological innovation, the artificial intelligence industry reduces the uncertainty caused by the introduction of innovative technology by accelerating the introduction of technology, which is conducive to reducing the risk of technological innovation investment and stimulating innovation efficiency^18,19^. At the same time, intelligent technology provides a new driving force for innovation efficiency, which is based on information flow, effectively integrates technology, capital, manpower and material, and deeply integrates with cloud computing, Internet and other industries, effectively improving management innovation, institutional innovation and model innovation. From the perspective of service innovation, knowledge and technology innovation and product innovation, collaborative innovation efficiency can be achieved.

This study suggests that the artificial intelligence sector has a substantial influence on the advancement of high-quality economy. However, it also indicates that China’s current level of development in this industry is not sufficiently high and there is still ample room for enhancement. The future establishment of the artificial intelligence industry can be initiated from the following perspectives. 1. It is important to enhance the development and funding of the artificial intelligence sector. There is a need to continuously diversify, broaden, and innovate the range of services offered by artificial intelligence and enhance the quality of intelligent services. Efforts should be made to expand the artificial intelligence industry and establish a cluster of intelligent industrial chains. Furthermore, increasing investment in advanced artificial intelligence technology and application production will contribute to the growth of this industry. 2. It is essential to establish policies that support the growth of the artificial intelligence industry within the framework of advancing the national economy. These policies should leverage the strengths of private capital and drive forward the high-quality development of artificial intelligence. 3. Rebalance the distribution of resources in the artificial intelligence industry. There is a noticeable disparity in resource allocation between the eastern, central, and western regions in the development of the artificial intelligence industry. It is important to enhance the competitive advantage of the artificial intelligence industry in the eastern coastal areas while also promoting its growth in the central and western regions. Efforts should be made to encourage the flow of artificial intelligence industry resources and national policies to these regions, thereby improving overall resource allocation levels within the industry.

The artificial intelligence industry improves its innovation ability by promoting the transformation of intelligent technologies. The artificial intelligence industry promotes the transformation of intellectual energy technology, which can well drive the development of a series of related industries, promote industrial division of labor, coordination and development, and thus form industrial innovation cooperation^22,23^. At the same time, the artificial intelligence industry has formed full innovation cooperation, making enterprises, government organizations, service agencies, social groups and other organizations jointly build an innovation network system with the artificial intelligence industry as the core. For example, Weibo, wechat and other social platforms can spread and share news quickly through a huge consumer group, so the greater the network value created, the more enterprises and consumers can be attracted to enter. Based on the transformation and application of intelligent technology, the artificial intelligence industry has improved its innovation ability and driven high-quality economic growth through cooperation between different industrial chains^24,25^.

### Encourage the advancement of the strategy involving “artificial intelligence + X” and enhance the overall economic competitiveness of the area

This paper demonstrates that the artificial intelligence industry is an important driving force that cannot be ignored for high-quality economic growth, with direct and indirect impacts. To promote the development of the “artificial intelligence + X” strategy and improve the overall level of regional economy, we should pay attention to the following aspects^26–31^: 1. Strengthen the intelligent, intensive and collaborative characteristics of artificial intelligence, build a more efficient, convenient and flexible “artificial intelligence + X” regional intelligent innovation model, and maximize the competitive advantage of the artificial intelligence industry. 2. Develop a patent protection system for artificial intelligence technology. The lack of relevant laws and regulations on the protection of artificial intelligence technology patent achievements, the state should strengthen the protection of artificial intelligence industry patents, and create a good development environment for the development of artificial intelligence industry. 3. Build an intelligence sharing service platform to provide intelligent services. Through the construction of an intelligent sharing service platform integrating government, universities and enterprises, new knowledge, new technology, massive information, big data and other new production factors are shared on the intelligent service platform to form an intelligent sharing platform in characteristic fields and provide intelligent services in an all-round way, thus improving regional economic strength.

### Promote differentiated intelligent technology support and balance the regional differences in the distribution of artificial intelligence industry resources

This study reveals that there is a clear imbalance in the spatial distribution of the artificial intelligence industry’s development level. This suggests that the “artificial intelligence +” strategy is not fixed and unchangeable, and it is important to implement dynamic and differentiated intelligent technology to address regional disparities in the distribution of artificial intelligence industry resources. Additionally, there are significant regional differences in the role of the artificial intelligence industry in promoting high-quality economic development. The eastern region, in particular, has higher requirements for the development^32,33^. The artificial intelligence industry in the central and western regions lags behind that in the eastern regions. It is important to boost the development of artificial intelligence industry in these areas, narrow the economic gap with developed regions, and achieve high-quality and balanced economic growth. Additionally, it is crucial for the government to consider the actual situation and empirical laws of artificial intelligence industry development when implementing industrial policies, as this will enhance the competitiveness of artificial intelligence technology in regional economic development and effectively promote high-quality economic growth through human intelligence.

## Conclusions

The impact of the artificial intelligence industry on high-quality economic development is intricate. This paper examines the artificial intelligence industry within the framework of a high-quality economic development system, based on the concept of new development. By analyzing its positive effects on economic innovation, coordination, environmental sustainability, openness and sharing from economic, environmental and social perspectives, this paper aims to understand the mechanism effect of artificial intelligence industry on high-quality economic development. Therefore, enhancing the development of the artificial intelligence industrial system will contribute to advancing high-quality economic growth. Research on the impact of the artificial intelligence industry on high-quality economic development yields diverse results. Specifically, during the period of 2008–2012, when the economy was still in a phase of rapid growth, the influence of the artificial intelligence industry on high-quality economic development was not very apparent; however, from 2012 to 2017, as the economy transitioned into a stage of high-quality growth, it became evident that the artificial intelligence industry played a significant role in promoting such growth. Rephrased: The impact of the artificial intelligence industry on the high-quality development of the eastern, central, and western regions varies significantly based on regional distribution. This discrepancy is attributed to varying levels of high-quality economic development in these regions, with the rapid growth of the artificial intelligence industry in the eastern region having a more pronounced effect on high-quality economic development. Conversely, the impact of the artificial intelligence industry in the central and western regions on high-quality economic development is relatively weak.

## Data Availability

The experimental data used to support the findings of this study are available from the corresponding author on request.
