# Author Correction: The influence of AI on the economic growth of different regions in China

**DOI:** 10.1038/s41598-024-60642-1

**Published:** 2024-04-29

**Authors:** Shuang Lin, Minke Wang, Chongyi Jing, Shengda Zhang, Jiuhao Chen, Rui Liu

**Affiliations:** 1 School of Economics and Management, Civil Aviation Flight University of China, Deyang, 618307 China; 2 School of Airport Engineering, Civil Aviation Flight University of China, Deyang, 618307 China; 3 Department of Administration, Chengdu University of TCM, Chengdu, 611137 China

Correction to: *Scientific Reports* 10.1038/s41598-024-59968-7, published online 22 April 2024

The Funding section in the original version of this Article was incomplete.

“This research is funded by China Scholarship Council and the local innovation sub-project of the Western Project of the Sichuan Provincial (Grant No: 202108510117).”

now reads:

“This research is funded by the China Scholarship Council and the local innovation sub-project of the Western Project of the Sichuan Provincial (Grant No: 202108510117). It is supported by the Sichuan Provincial Science Foundation Project (Grant No: 2022NSFSC0928).”

The original Article has been corrected.

